# Awareness and use of folic acid among women of childbearing age in Benue State, Nigeria

**DOI:** 10.11604/pamj.2020.37.60.22848

**Published:** 2020-09-15

**Authors:** Ubong Akpan Okon, Baffa Sule Ibrahim, Rabi Usman, Elizabeth Adedire, Muhammad Shakir Balogun, Adebola Olayinka

**Affiliations:** 1 Nigeria Field Epidemiology and Laboratory Training Program, Abuja, Nigeria,; 2 Africa Field Epidemiology Network, Kampala, Uganda,; 3 University of Maryland Baltimore, Nigeria Program, Nigeria,; 4 World Health Organization, Abuja, Nigeria.

**Keywords:** Folic acid, neural tube defects, peri-conceptional, childbearing age

## Abstract

**Introduction:**

every year in Nigeria 12,695 babies are born with neural tube defects. Folic acid reduces the risk of neural tube defect by about 50% among peri-conceptional users. We conducted this study to determine the awareness and use of folic acid among women of childbearing age in Benue State, Nigeria.

**Methods:**

we conducted a cross-sectional study among women attending selected among six secondary health facilities in Benue State using a multi-stage sampling technique. We interviewed 586 women aged 15-49 years using structured questionnaires to obtain information on awareness and use of folic acid. We performed univariate, bivariate and multivariate analysis at 5% significance.

**Results:**

interviewed 586 women with the mean age 27 ± 6.9 years; 281(48%) were aware of folic acid as a supplement, 178 (30.4%) knew the dietary source of folic acid while 152 (26%) knew the benefit of folic acid. The commonest source of information was health professionals [195 (51%)]. Only 221 (37.7%) used folic acid, 14 (1.7%) of them using it within the first trimester. The commonest reason why women did not take folic acid as supplement was unpleasant smell [124 (21%)]. Awareness of folic acid benefit (OR: 6, 95%CI = 3.9-8.8), level of education (OR: 2.0, 95%CI = 1.2-3.3) and employment status (OR: 1.6, 95%CI= 1.1-22) were significantly associated with folic acid use. Awareness of folic acid use was an independent predictor of folic acid use (AOR: 7.9 95%Cl: 5.3-11.7).

**Conclusion:**

awareness and use of folic acid among women of childbearing age was low. Awareness is a predictor of folic acid use. We recommend the promotion of awareness and use of folic acid in pregnant women.

## Introduction

Folic acid is an essential vitamin sometimes referred to as vitamin B9 [1]. It is highly recommended in pregnancy especially before and during the peri-conceptional period since it cannot be produced by the body [2]. It is found in green leafy vegetables, broccoli, peas, grains and cereals. It is known as folate in folic acid fortified food. Folic acid plays significant roles in the synthesis of DNA, and consequently cell division which is essential for cell growth and differentiation and is important in cell structure, cell signaling, protein translation, enzymes, catalytic enzyme sites, and enzymatic reactions [3]. These processes are critical for organ development in the fetus which occurs during organogenesis during the 4^th^ - 8^th^ weeks of gestation. Studies have shown that deficiency of folic acid in pregnancy have been linked to birth defect [3]. The most common birth defect associated with folic acid deficiency is the neural tube defect. The neural tube is the embryonic precursor to the brain and spinal column. Neural tube defect occurs when the neural tube fails to close between 21 and 28 days after conception even before the women realize they are pregnant [4]. This error could lead to death or permanent damage to the brain, spinal cord and spinal nerves, thus leading to multiple lifelong disabilities such as lower limb paralysis, bowel and bladder incontinence, hydrocephalus, intellectual and learning disabilities [4]. Neural tube defects (NTDs) comprise spine bifida, encephalocele, anencephaly, diastematomyelia, various tethered cord syndromes, syringomyelia and lipoma of the conus medullaris [5].

In Nigeria, the neonatal mortality rate is 37 deaths per 1,000 live births and the perinatal mortality rate is 41 per 1,000 pregnancies [6]. Neural tube defect contributes to the increase in neonatal and perinatal mortality. March of Dimes estimated that approximately 12, 000 births are affected each year in Nigeria, one of the highest in Africa [7]. Studies indicate that the incidence of NTDs in northcentral Nigeria was 0.5 per 1000 live births in 2014 and 22.5% in southwestern Nigeria in 1997 [8,9]. Neural tube defect is highly preventable through adequate intake of folic acid supplement and food fortified with folic acid [7]. It is well-documented that folate intake during pre-conceptional period and early during pregnancy significantly reduces the risk of having birth defects by 50 - 70% [10]. Folic acid also prevents congenital heart defects, growth retardation, low-birth weight, smaller head and chest circumference, preterm delivery and cleft lip [11]. The Centers for Disease Control and Prevention (CDC) recommends that all women of childbearing age consume 0.4mg of folic acid per day and 5 mg for women at higher risk of neural tube defects [12]. In Nigeria women are given 0.4mg of folic acid during antenatal care as a routine regimen; however, a study in Plateau State in northcentral Nigeria has shown a significant decline in the knowledge and use of folic acid among pregnant women visiting antenatal care which could be a true reflection of the state of knowledge and use of folic acid among pregnant women on antenatal in Benue State since all share similar cultures and norms [13]. However, there are no studies on the awareness and use of folic acid among women of childbearing age who are not pregnant. To address this gap, we therefore conducted this study to determine the awareness and use of folic acid among women of childbearing age in Benue State, Nigeria.

## Methods

This study was conducted in Benue State Nigeria which is located in north central Nigeria and has a population of about 5,741,800 according to 2016 projected population [6]. The state is divided into three zones - A, B and C - with 23 Local Government Areas (LGA). Zones A and B comprise 7 LGAs each while Zone C has 9. We conducted a descriptive cross-sectional study over a 6-month period among women of childbearing age between the age of 15 and 49 years who attended selected facilities and are residents in Benue State. The sample size was calculated using the formula for sample size determination [14].

$$n=\frac{\left(z^{2}pq\right)}{d^{2}}$$

Where: z = 1.96, p = the proportion of women with a good knowledge of folic acid from previous study 65% [8], q = complementary probability of p = 1 - p and d = precision here set at 4%.

$$sample\text{ size =}\frac{\left(1.92^{2}\times 0.65\times 0.35\right)}{{\left(0.04\right)}^{2}}$$

= 546

We included a non-response rate of 10%. Sample size x 1/(1-nr). Assuming nr of 10%

$$=546\times \frac{1}{\left(1-0.1\right)}$$

**Figure 1:**
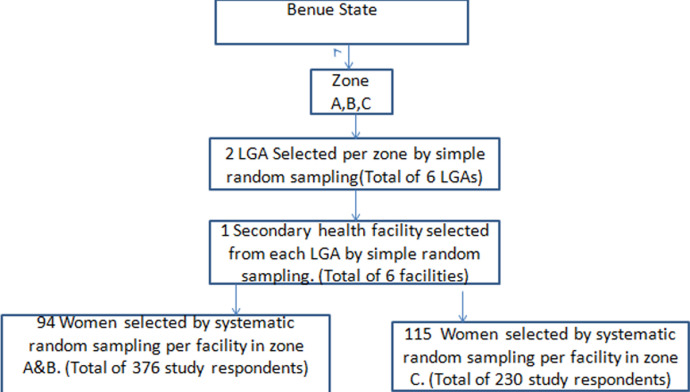
flowchart of recruitment of study respondents

**Sampling technique:** a multistage sampling technique was used to select respondents and health facilities for the study. First, two LGAs were selected from each zone using a simple random sampling technique. Then, one secondary facility was selected from each LGA using a simple random sampling technique. At the third stage, participants were selected with probability proportionate to the size of the population using a systematic random sampling. One hundred and eighty-eight participants were selected each from zone A and B while two hundred and thirty participants were selected from zone C (Figure 1).

**Data collection/analysis:** a semi-structured interviewer-administered questionnaire was administered in selected health facilities. The questionnaire contained questions on demographic and socioeconomic characteristics such as age, occupational status, educational status, economic status, plan for pregnancy and parity, awareness, source of knowledge, use of folic acid supplements, fortified foods, and putative predictors of folic acid use. The questionnaire was coded and pre-tested. A total of six research assistants were trained on how to administer the questionnaire and deployed to the selected facilities. Data was entered in Microsoft Excel, cleaned, and analyzed using Epi Info 7™. The participant was considered to have knowledge of the dietary source if he/she agrees to know other sources of folic acid and could identify at least one dietary source of folic acid. Univariate and bivariate analysis was done to determine odds ratios at 95% confidence interval. Logistic regression was done to control for confounders.

**Ethical considerations:** ethical approval was obtained from the Benue State Ministry of Health Human Research Ethics Committee. Permission was also obtained from the health facilities where the respondents were recruited. Informed oral consent was obtained from individual participants. Each respondent´s autonomy and rights to their own decisions and beliefs were respected as their participation was voluntary without fear of harm. All data collected from respondents were handled with utmost confidentiality.

## Results

**Figure 2:**
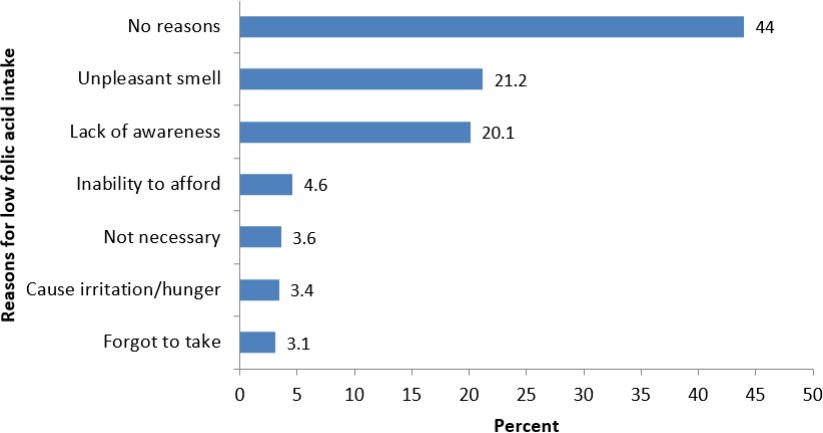
reasons for lack of folic acid use among women of childbearing age in Benue State, 2016

A total of 606 women were selected out of which 20 women declined interview, giving a response rate of 97%. Their mean age was 27±6.9 years. Those aged 20-24 years accounted for 33.3% of respondents. Married and educated respondents were 295 (50.3%) and 539 (91.9%) respectively (Table 1). Two hundred and eighty-one (48%) respondents were aware of folic acid as a supplement; 178 (30.4%) of the respondents knew the dietary sources of folic acid (Table 2) while 152 (26%) knew the problems associated with folic acid deficiency (Table 2). The sources of information of folic acid among the respondents were health professionals (51%), media (14%) and friends/relations (5.8%) (Table 2). Only 221 (37.7%) used folic acid, out of which 94 (42.5%) took folic acid after the first trimester of pregnancy while 14 (1.7%) took folic acid within the first trimester of pregnancy (Table 2). Reasons reported by the respondents on why women do not use folic acid were: no reasons (44%), and unpleasant smell (21.2%) (Figure 2). Bivariate analysis showed that women who were aware of folic acid as a supplement were more likely to take folic acid supplement than women who are unaware of folic acid (OR: 6.0, 95%Cl: 3.9-8.8) (Table 3). Uneducated women were more likely not to take folic acid than educated women (OR: 2.0 95%Cl: 1.2-3.3) and those who were employed were more likely to take folic acid. (OR: 1.6 95%Cl: 1.1-2.2) (Table 3). However, after multivariable logistic regression, only awareness of folic acid use was found to be an independent predictor of folic acid use among women of childbearing age (AOR: 7.9 95%Cl: 5.3-11.7) (Table 4).

**Table 1:** socio-demographic characteristics of women of childbearing age in Benue Nigeria-2016

Socio-demographic Characteristics n=581	Frequency (%)
**Age group**	
15-19	37 (6.3)
20-24	195 (33.3)
25-29	167 (28.5)
30-34	102(17.4)
35-39	37(6.3)
40-44	30(5.1)
45-49	18(3.1)
**Ethnicity**	
Etulo	7(1.2)
Igede	27(4.6)
Others	90(15.4)
Idoma	170(29.0)
Tiv	292(49.8)
**Marital Status**	
Single	291(49.7)
Married	295(50.3)
**Education Status**	
Educated	539(91.9)
Uneducated	47(8.0)
**Employment Status**	
Unemployed	309(52.7)
Employed	277(47.3)
**Parity (Previous birth)**	
≤1	373(63.6)
≥2	213(36.4)
**Income Level($)(n=263)**	
≤66	96(16.4)
67-131	83(14.2)
132-226	45(7.7)
227-325	19(3.2)
326-489	7(1.2)
≥490	13(2.2)

**Table 2:** univariate analysis of outcome variables of folic acid awareness and use among women of childbearing age, Benue State, Nigeria-2016

Variables	Frequency (%)
**Awareness of folic acid supplement (n=586)**	
Yes	281 (47.9)
No	305 (52.1)
**Awareness of dietary sources of FA (n=586)**	
Yes	178(30.4)
No	408(69.6)
**Knowledge problem associated with FA deficiency (n=586)**	
Goiter	1(0.2)
Blindness	2(0.3)
Arthritis	9(1.5)
Weakness of the bone/teeth	40(6.8)
Anaemia	54(9.2)
*Birth defect/neural tube defect	152(25.9)
I don’t know	328(55.9)
**Folic Acid Use(n=586)**	
Yes	221(37.7)
No	365(62.3)
**Period of FA Use (n=221)**	
Within First Trimester	4(1.8)
Before Pregnancy	61(27.6)
After First Trimester	96(43.3)
Can’t Remember	60(27.1)
**Sources of Information (n=281)**	
Friends/Relatives	22(7.8)
Media	42(14.9)
Healthcare Professionals	180(64.1)
Can’t Remember	37(13.2)

**Table 3:** bivariate analysis of factors associated with low folic acid use among women of childbearing age, Benue State, 2016

Variable	Use of Folic acid (Frequency)		Odds Ratio (95% CI)
	Yes	No	
**Awareness of folic acid**			
Yes	172	109	6.0 (3.9-8.8)
No	49	256	
**Marital Status**			
Single	99	298	1.3 (0.98-1.9)
Married	122	75	
**Educational Status**			
Educated	196	290	2.0 (1.2-3.3)
Uneducated	25	75	
**Occupational status**			
Employed	119	158	1.6 (1.1-2.2)
Unemployed	102	207	
**Parity (Previous birth)**			
≤1	175	198	1.1 (0.8-1.6)
≥2	106	107	

**Table 4:** multivariable logistic regression of factors associated with low folic acid use among women of childbearing age, Benue State, 2016

Variable	Use of Folic acid (Frequency)		Adjusted Odds Ratio (95% CI)
	Yes	No	
**Awareness of folic acid**			
Yes	172	109	7.9 (5.3-11.7)
No	49	256	
**Educational Status**			
Educated	196	290	1.9 (0.9-4.3)
Uneducated	25	75	
**Occupational status**			
Employed	119	158	1.4 (0.9-2.0)
Unemployed	102	207	

## Discussion

Our study showed that the level of awareness of folic acid among women of childbearing age in Benue State was low. Our findings are similar to the results reported among women in China (36.0%) [15]. It is remarkable that less than half of the pregnant women were aware of folic acid as a vitamin supplement. This level of folic acid awareness is less than what was reported from Jos in Nigeria (64%), Turkey (63.2%), and Qatar (53.7%) [8, 16, 17] but higher than the proportion from China (36.0%) [15]. This low rate of awareness among the study population could be as a result of low intake of folic acid supplements among pregnant women as seen in this study and probably low antenatal clinic attendance since the most common source of information on folic acid was through health workers, as folic acid is usually prescribed routinely in addition to iron supplements during pregnancy in this country mainly for the prevention of anemia. Hence, if the pregnant women were not accessing antenatal care, they were not likely to have heard about the importance of folic acid use in pregnancy. It is to be expected that the proportion of respondents who understood the benefits of folic acid should be lower still. And this is comparable to the finding of the Jos study in which 26.9% of pregnant women were aware of the role of folic acid in the prevention of NTDs [8].

Awareness about the benefits of folic acid is similarly low in other parts of Nigeria: 25.5% and 24.1% were reported from Ido-Ekiti and Port Harcourt respectively [18, 19]. This low level of awareness of the benefits of folic acid, even though the majority of respondents were educated, is worrisome as lack of awareness of the role of folic acid in the prevention of NTDs would lead to low usage which would in turn increase the risk of NTDs. This emphasizes the need for health education targeting women of reproductive age on the importance of folic acid as an important supplement in our environment especially during antenatal care visits. The low level of awareness is unsurprisingly attended by a low peri-conceptional intake of folic acid with only 1.7% of them taking it within the first trimester. Similar findings have been reported in other countries [20]. Most of the respondents attributed their lack of folic acid use to no reasons at all but one-fifth mentioned the unpleasant taste of folic acid as a reason for not using it. There is a need to encourage pregnant women to use folic acid by improving the taste of folic acid tablets by pharmaceutical companies. This also underscores the need for the fortification of our staple food stuff such as flour, sugar and salt with folic acid with the aim of increasing its intake among women of reproductive age in our setting. This strategy has been shown to be effective in reducing incidences of NTDs in developed countries [20, 21].

The main source of information about folic acid among the women was through the health workers, similar to the study in Jos, but in contrast to findings from elsewhere where the media was the main source of information about folic acid [13]. This may not be unconnected to the fact that most of the women were multiparous and so were at one time or the other in contact with these health workers and or were prescribed the vitamin supplement during their previous pregnancies. More efforts are therefore needed to promote awareness about folic acid through the media because of its wider coverage among the population. Based on our finding that the awareness of folic acid is an independent predictor of folic acid use among women of childbearing age, we recommend interventions to increase awareness of folic acid use, its benefits and its role in preventing NTDs which are expected to translate to increased intake of peri-conceptional folic acid. However, this study is not without limitations. Findings from hospital-based studies may not be truly generalizable to the larger community. All responses were self-reported and may be subject to recall bias which we tried to mitigate by restricting questions to events that occurred within the last six months prior to the interview.

## Conclusion

Awareness and use of folic acid were significantly low among women of childbearing age in Benue State. Lack of awareness is an independent predictor of low folic acid use among women of childbearing age in Benue State.

### What is known about this topic

- Folic acid used during pre-conception prevent neural tube defect;
- Awareness of folic acid the role of folic acid is low in other region of Nigeria.

### What this study adds

- The study added that awareness and pre-conceptional use of folic acid is lower than the other states within the north central region;
- Awareness of folic acid use is an independent predictor of folic acid use among women of childbearing age.
